# Breast Sarcomas, Phyllodes Tumors, and Desmoid Tumors: Turning the Magnifying Glass on Rare and Aggressive Entities

**DOI:** 10.3390/cancers15153933

**Published:** 2023-08-02

**Authors:** Miguel Esperança-Martins, Cecília Melo-Alvim, Sara Dâmaso, Raquel Lopes-Brás, Tânia Peniche, Gonçalo Nogueira-Costa, Catarina Abreu, Helena Luna Pais, Rita Teixeira de Sousa, Sofia Torres, Lina Marcela Gallego-Paez, Marta Martins, Leonor Ribeiro, Luís Costa

**Affiliations:** 1 Medical Oncology Department, Centro Hospitalar Universitário Lisboa Norte, 1649-028 Lisboa, Portugal; cecilia.alvim.moreira@gmail.com (C.M.-A.); sarafdamaso@gmail.com (S.D.); raquellopesbras@gmail.com (R.L.-B.); g.nogueiradacosta@gmail.com (G.N.-C.); catarinaabreupm@gmail.com (C.A.); helenalunapais@gmail.com (H.L.P.); arita.sousa@gmail.com (R.T.d.S.); sofiac.torres@gmail.com (S.T.); lcabreuribeiro@gmail.com (L.R.); 2 Luis Costa Lab, Instituto de Medicina Molecular João Lobo Antunes, Faculdade de Medicina da Universidade de Lisboa, 1649-028 Lisboa, Portugal; t.peniche@campus.fct.unl.pt (T.P.); linagallego@medicina.ulisboa.pt (L.M.G.-P.); marta.martins@medicina.ulisboa.pt (M.M.); 3 Faculdade de Medicina da Universidade de Lisboa, 1649-028 Lisboa, Portugal

**Keywords:** breast sarcoma, soft tissue sarcoma, breast angiosarcoma, breast liposarcoma, breast leiomyosarcoma, breast rhabdomyosarcoma, breast chondrosarcoma, breast phyllodes tumor, breast desmoid tumor

## Abstract

**Simple Summary:**

Breast sarcomas, phyllodes tumors, and desmoid tumors are rare and unique entities whose epidemiologic, molecular (especially genomic), clinical, prognostic and predictive, and therapeutic landscapes are poorly characterized. Despite their rarity, the potential aggressiveness and significant functional impact of these entities make it important to characterize them thoroughly. The challenges of mapping the genomic landscape of breast sarcomas for the first time and updating the existing literature in terms of risk, prognostic and predictive factors, and novel treatment options motivated us to pursue our goal of producing a high-quality review focused on this group of neoplasms. This article intends to be a new source of data for researchers seeking new targets to address therapeutically and a tool that can be used by clinicians to guide their decisions when treating patients with breast sarcomas, phyllodes tumors, and desmoid tumors.

**Abstract:**

Breast sarcomas (BSs), phyllodes tumors (PTs), and desmoid tumors (DTs) are rare entities that arise from connective tissue. BSs can be classified as either primary or secondary, whether they develop de novo or after radiation exposure or lymphedema. PIK3CA seems to play an important common role in different BS. Malignant PTs show similar behavior to BSs, while DTs are locally aggressive but rarely metastasize. BSs usually present as unilateral, painless, rapidly growing masses with rare nodal involvement. The diagnosis should be based on magnetic resonance imaging and a core needle biopsy. Staging should comprise a chest computed tomography (CT) scan (except for benign PT and DT), while abdominal and pelvic CT scans and bone scans should be added in certain subtypes. The mainstay of treatment for localized BS is surgery, with margin goals that vary according to subtype. Radiotherapy and chemotherapy can be used as neoadjuvant or adjuvant approaches, but their use in these settings is not standard. Advanced BS should be treated with systemic therapy, consistent with recommendations for advanced soft tissue sarcomas of other topographies. Given the rarity and heterogeneity of these entities, multidisciplinary and multi-institutional collaboration and treatment at reference centers are critical.

## 1. Introduction

Breast cancer is one of the most commonly diagnosed cancers worldwide [1]. The vast majority of cases correspond to breast carcinomas arising from epithelial tissue, with breast sarcomas, which arise from stromal cells and connective tissue, accounting for less than 1% of all breast neoplasms and less than 5% of all soft tissue sarcomas (STS) [1,2]. 

The authors performed a comprehensive review of breast sarcomas, phyllodes tumors, and desmoid tumors, addressing their epidemiology, prevention and screening strategies, genomic profile and molecular landscape, clinical features, diagnosis, staging, prognostic and predictive factors, and treatment strategies. 

## 2. Epidemiology and Risk Factors

Breast sarcomas are particularly rare entities. Of the 27,881 malignant breast tumors diagnosed at the Mayo Clinic between 1940 and 1999, only 0.0006% were breast sarcomas [3]. Data from the Surveillance, Epidemiology, and End Results (SEER) program of the National Cancer Institute (NCI) showed an annual incidence of breast sarcoma of 4.6 cases per 1 million women [4]. Between 1 and 2% of cases occur in men [2], and the median age at diagnosis ranges from 47 to 50 years [2].

Breast sarcomas can be conceptually divided into primary and secondary sarcomas [2]. Primary breast sarcomas typically arise de novo from the breast parenchyma with no known specific risk factors, except for rare genetic syndromes, such as Li–Fraumeni syndrome, hereditary retinoblastoma, familial polyposis, or neurofibromatosis type 1, and certain environmental exposures, such as phenoxyacetic acid herbicides, which have been associated with an increased risk of STS in general [2]. In addition, other environmental exposures such as arsenic compounds, vinyl chloride, various types of immunosuppressive agents, the human immunodeficiency virus (HIV), and human herpes virus 8 (HHV-8) may also be associated with an increased risk of sarcomas [2]. Primary breast sarcomas/connective tissue neoplasms are histologically heterogeneous, with examples of malignant phyllodes tumors, angiosarcomas, desmoid tumors, liposarcomas, leiomyosarcomas, and rhabdomyosarcomas [2,5]. Secondary breast sarcomas typically develop after breast or chest wall irradiation. To be considered radiation-related, a secondary breast sarcoma must develop within the previously irradiated area, be of a different histology than the original tumor, and occur after a long latency period—more than 4 years by Cahan’s original definition or more than 2 years by other modified definitions [2]. Women who have been previously irradiated as part of treatment for in situ or invasive breast cancer or individuals who have been previously irradiated as part of treatment for Hodgkin’s lymphoma are most commonly affected [2]. In addition to radiation, an association between chronic lymphedema and angiosarcoma was first described by Stewart and Treves in 1948 (specifically, they reported six cases of patients who initially presented with lymphedema following mastectomy and axillary dissection and who subsequently developed angiosarcoma) and later supported by a number of studies that have helped to strengthen this conceptual link [2]. Typically, lymphedema-associated angiosarcomas present between 8 and 24 years after the development of lymphedema and show significant clinical aggressiveness and a poor prognosis [6]. However, lymphedema-associated angiosarcomas are much rarer than radiation-associated sarcomas, with most patients with secondary breast sarcomas presenting with the latter [2]. 

A recent retrospective study that included two cohorts of US breast cancer survivors (the Kaiser Permanente [KP] cohort and the SEER 13 registry cohort) investigated potential co-factors for the development of treatment-related thoracic STS [7]. In the KP cohort, 19 (0.1%) of 15,940 eligible, evaluable women developed thoracic STS (eleven angiosarcomas and eight other subtypes) [7]. The vast majority (94.7%; 18 of 19) of thoracic STS occurred in women treated with radiotherapy, showing that radiotherapy was associated with a significantly increased risk of developing thoracic STS (relative risk [RR] 8.1; *p* = 0.0052) [7]. However, there was no association with the prescribed dose, fractionation, or boost. The RR of angiosarcoma after anthracyclines was 3.6 (*p* = 0.058), while alkylating agents were associated with an increased risk of developing other sarcomas (RR 7.7; *p* = 0.026) [7]. Interestingly, a history of hypertension (RR 4.8; *p* = 0.017) and diabetes (RR 5.3; *p* = 0.036) were each associated with an approximately five-fold higher risk of angiosarcoma [7]. These are novel findings that warrant further investigation, as hypertension and diabetes are potential targets for preventive strategies and increased surveillance [7]. In the SEER cohort, 430 (0.1%) of 457,300 patients subsequently developed thoracic STS (268 angiosarcomas and 162 other subtypes) [7]. Most cases (77.9%; 335 of 430) occurred after radiotherapy, which was associated with a significantly increased risk of developing thoracic STS (RR 3.0; *p* < 0.0001). For angiosarcoma, the RR for breast-conserving surgery plus radiotherapy versus mastectomy plus radiotherapy was 1.9 (*p* = 0.012) [7]. Up to 10 years after radiotherapy, the cumulative incidence of thoracic STS was 0.21% in the KP cohort and 0.15% in SEER [7].

Angiosarcomas are consistently reported as the most common histologic subtype of secondary breast sarcomas [2,5]. 

Patients with secondary breast sarcoma tend to be older—probably reflecting the older average age of breast cancer patients—than patients with primary breast sarcoma [2]. 

## 3. Prevention and Screening

### 3.1. Prevention

Of the several risk factors previously mentioned, some are modifiable, and avoiding them may reduce the risk of developing breast sarcoma. 

It is not clear whether newer radiotherapy techniques, such as intensity-modulated radiotherapy or hypofractionated regimens, can reduce this risk. In cases where there is a known increased risk of developing sarcoma (such as inherited mutations of the ataxia telangiectasia mutated [*ATM*] gene), women may choose to have a mastectomy instead of breast-conserving surgery to avoid adjuvant radiotherapy. Similarly, breast cancer patients with documented germline mutations in both *TP53* and *ATM* should not be treated with radiotherapy.

Limiting or eliminating exposure to environmental factors, such as phenoxyacetic acid herbicides, arsenic compounds, and vinyl chloride, may also contribute to an overall reduction in the risk of developing sarcoma. 

### 3.2. Screening

Some genetic syndromes may predispose to an increased risk of STS [2]. Li–Fraumeni syndrome, caused by a heterozygous germline pathogenic variant in the *TP53* gene, is one of these syndromes. Evidence suggests that a specific surveillance strategy for Li–Fraumeni cases could improve the 5-year survival rate of individuals undergoing surveillance compared to those not undergoing surveillance [8]. Annual breast magnetic resonance imaging (MRI) may be alternated with whole-body MRI every 6 months, as recommended in the latest clinical guidelines of the Sociedad Española de Oncología Médica (SEOM) [8]. The use of mammography for screening purposes is not recommended in these patients to avoid radiation exposure [8]. This surveillance strategy is recommended for patients harboring a *TP53* likely pathogenic or pathogenic variant and should be initiated as soon as this variant is detected [8]. These individuals should also be encouraged to adopt a healthy lifestyle (avoid smoking and prolonged and unprotected exposure to known carcinogens, eat a healthy diet, and exercise frequently) [8]. 

Given the very low incidence and exceeding rarity of breast sarcoma, there is no specific screening strategy for these tumors in individuals with no known hereditary risk. In this population, the screening strategy for breast neoplasms should follow the same recommendations proposed for the average-risk population, using mammography. However, it should be emphasized that this screening strategy is not intended to screen for or diagnose breast sarcomas (but rather breast carcinomas) and that the use of mammography as a screening method may fail to identify these tumors, as explained in Section 6 (diagnosis and staging). Therefore, breast MRI is the preferred method for diagnosing these mesenchymal neoplasms. General recommendations for breast cancer screening may vary from organization to organization (Table 1).

## 4. Genomic Profile and Molecular Landscape of Breast Sarcomas

For the purpose of this review, cBioPortal was used to access various sarcoma cohorts and subsequently identify those that included breast sarcoma cases [11,12]. Three cohorts containing genomic and clinical data on breast sarcomas were selected, and the data were subsequently downloaded from the cBioPortal online repository:

- Cohort A, from the Angiosarcoma Project [13], included data on whole exome sequencing (WES), medical records (radiation exposure prior to sarcoma diagnosis, type and duration of adjuvant treatments, etc.), and pathology results and patient-reported data [14]. Although this cohort included a total of 83 samples, only 29 were from the breast.
  ○ Cohort A.1 included samples from patients who had been exposed to radiation prior to a sarcoma diagnosis.
  ○ Cohort A.2 included samples from patients who had not been exposed to radiation prior to a sarcoma diagnosis.
- Cohort B, also from the Angiosarcoma Project [13], included data on WES, medical records, and pathology results and patient-reported data [15]. This cohort contained 48 angiosarcoma samples, of which only 19 were from the breast.
- Cohort C, from the Memorial Sloan Kettering Cancer Center (MSKCC) [16] MSK-IMPACT, included genomic data obtained through a panel of 505 genes (MSK-IMPACT gene panel) and clinical data, including the origin of each sample (primary or metastatic lesion). This cohort comprised a total of 2138 samples, of which 31 were from breasts. The cohort included samples from angiosarcoma, undifferentiated pleomorphic sarcoma/malignant fibrous histiocytoma/high-grade spindle cell sarcoma, desmoid/aggressive fibromatosis, leiomyosarcoma, and round cell sarcoma.
  ○ Cohort C.1 included samples from patients with a diagnosis of breast angiosarcoma.
  ○ Cohort C.2 included samples from patients with a diagnosis of other types of breast sarcoma.

A total of seventy-nine samples of breast angiosarcoma and four samples of other breast sarcomas were analyzed, with angiosarcoma being the most common type of breast sarcoma found. 

R programming language version 4.2.3 and RStudio interface version 2023.03.1 + 446 [17] were used to analyze this cohort. The “*dplyr*” package version 1.1.2 [18] was used for data manipulation, and the “*Maftools*” package version 2.14.05 [19] was implemented for genomic data summary, analysis, annotation, and visualization in Mutation Annotation Format (MAF).

**Figure 1:**
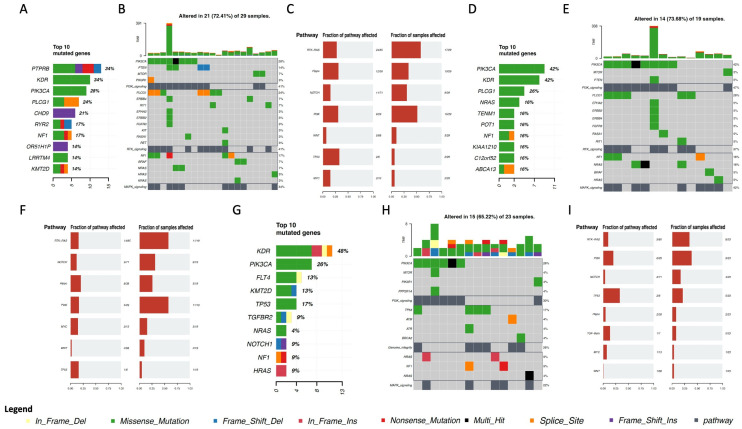
Genomic profile of angiosarcomas of the breast. (**A**) Top 10 mutated genes in cohort A; (**B**) Oncoplot for cohort A. Each row represents one gene, and each column represents one sample. The presence of alterations such as mutations is indicated by different colors, as explained in the legend (in-frame deletion, missense mutation, frame-shift deletion, in-frame insertion, nonsense mutation, multiple hit alterations, splice site mutation, frame-shift insertion, altered pathway). The overall dysregulation of specific pathways across different samples can be visualized. The percentage of samples affected is shown; (**C**) Enriched oncogenic signaling pathways in cohort A. The number of genes affected in the pathways and the number of samples with that pathway affected are shown; (**D**) Top 10 mutated genes in cohort B; (**E**) Oncoplot for cohort B; (**F**) Enriched oncogenic signaling pathways in cohort B; (**G**) Top 10 mutated genes in cohort C.1; (**H**) Oncoplot for cohort C.1; (**I**) Enriched oncogenic signaling pathways in cohort C.1.

In angiosarcomas, the most frequently mutated genes in all three cohorts were *KDR*, *PIK3CA*, and *NF1*, followed by *KMT2D*, *NRAS*, and *PLCG1*, which were among the top 10 genes in two of the three cohorts (Figure 1). This is particularly important because a panel of only 505 genes was used in cohort C, and thus some relevant genes may have been missed.

Interestingly, *PIK3CA* encodes a catalytic subunit of *PI3K*, a well-known gene implicated in the oncogenesis of several types of cancer and involved in lipid signaling, cell growth, proliferation, migration, and cell survival [20]. *PI3K* is currently a therapeutic target in some cancers, such as breast cancer [21]. *KDR*, also known as *VEGFR2*, plays a critical role in the angiogenic process [22], regulating endothelial cell proliferation, migration, and survival when activated by VEGF signaling [23]. *NF1* is a tumor suppressor gene that regulates cell growth and division and plays a role as a negative regulator of RAS activity [24]. *KMT2D*, also known as *MLL2*, is a histone methyltransferase involved in the epigenetic regulation of gene expression [25]. *NRAS* belongs to the RAS gene family and encodes an oncogenic GTPase involved in cell signaling [26]. *PLCG1* encodes an enzyme involved in intracellular signaling, playing a role in the phosphoinositide signaling pathway and regulating the production of the secondary messengers diacylglycerol and inositol triphosphate [27]. 

Notably, the most frequently mutated genes were shown to be involved in pathways known to be associated with cancer development, such as *RTK-RAS*, *PI3K*, *MAPK*, *NOTCH*, and *HIPPO* signaling (Figure 1B,C,E,F,H,I). 

**Figure 2:**
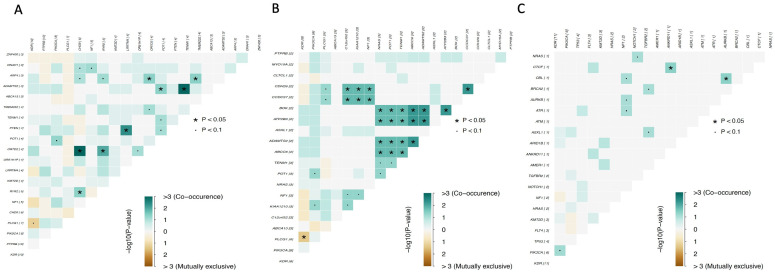
Mutually exclusive or co-occurring gene sets detected by pairwise Fisher’s exact test. (**A**) Cohort A; (**B**) Cohort B; (**C**) Cohort C.1. Color gradient represents the range of *p*-values (P) from Fisher’s exact test on a −log_10_ scale.

Analysis of the interactions between the 20 most frequently mutated genes showed that somatic mutations in *PLCG1* and *KDR* were mutually exclusive, whereas somatic mutations in *ABCC6*, *BOK*, *ATP2B3*, and *ADAMTS2* co-occurred (pairwise Fisher’s exact test *p* ≤ 0.05). The same was true for *ADAMTS2* and *TEMM1*, *PTEN and LRRTMY*, *OR7D2* and *RYR2*, and *CDH26* and *CCDC37* (Figure 2). 

**Figure 3:**
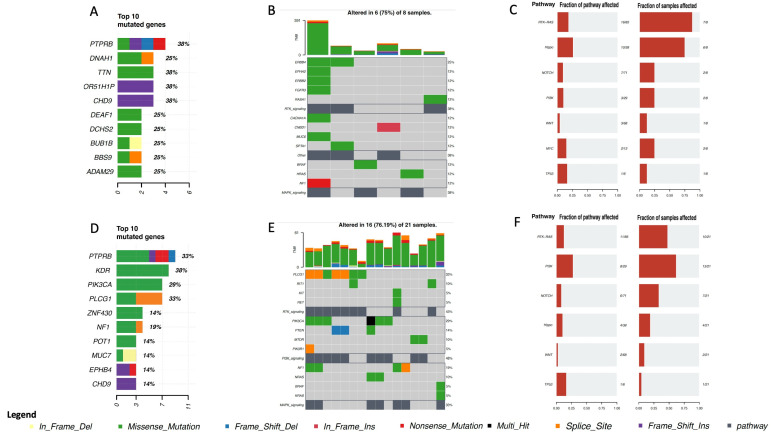
Genomic profile of signaling pathways in angiosarcomas of the breast that had been previously exposed to radiation versus non-exposed. (**A**) Top 10 mutated genes in cohort A.1; (**B**) Oncoplot for cohort A.1; (**C**) Oncogenic Signaling Pathways enriched in cohort A.1; (**D**) Top 10 mutated genes in cohort A.2; (**E**) Oncoplot for cohort A.2; (**F**) Enriched oncogenic signaling pathways in cohort A.2.

In cohort A, the samples were further subdivided according to whether patients had been exposed to radiation prior to sarcoma diagnosis (eight samples) or not (twenty-one samples). Analysis of the two groups showed that only two genes had mutations occurring in both: *PTPRB* and *CHD9* (Figure 3). *PTPRB* and *CHD9* are genes known to be associated with cancer. *PTPRB* is a receptor-type protein tyrosine phosphatase involved in the regulation of cellular signaling pathways and plays a role in cell adhesion and migration and in angiogenesis processes [28]; *CHD9* is involved in chromatin remodeling and gene expression regulation and belongs to the chromodomain helicase DNA-binding (CHD) protein family [29]. In addition, *DNAH1*, *OR51H1P*, *DEAF1*, *DCHS2*, *BUB1B*, *BBS9*, and *ADAM29* were the most frequently mutated genes in the group of patients previously exposed to radiation (Figure 3A), whereas *KDR*, *PIK3CA*, *PLCG1*, *ZNF430*, *NF1*, *POT1*, *MUC7*, and *EPHB4* were some of the most frequently mutated genes in the group of patients not previously exposed to radiation (Figure 3D). Although these genes seemed to distinguish the two cohorts, the pathways affected by their mutations were almost the same (Figure 3B,C,E,F). The most frequently affected pathways were *Hippo*, *Notch*, *PIK3*, *WNT*, *TP53*, and especially *RTK-RAS* (Figure 3).

**Figure 4:**
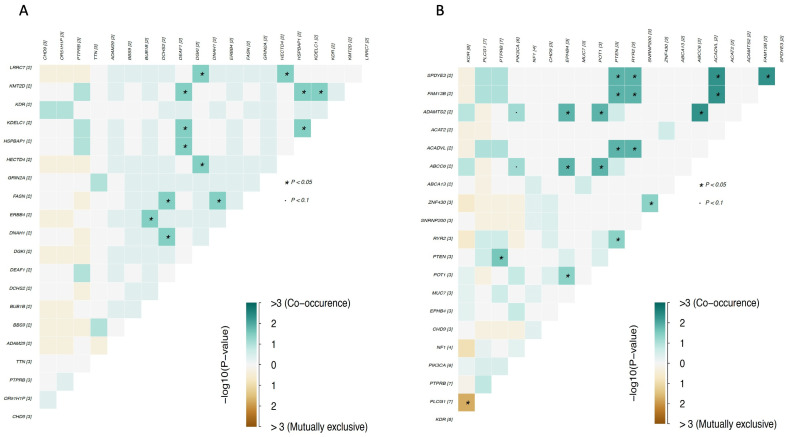
Mutually exclusive or co-occurring gene sets detected by pairwise Fisher’s exact test. (**A**) Plot for cohort A.1; (**B**) Plot for cohort A.2.

In the group of patients previously exposed to radiation, mutations in *DNAH1*, *DCHS2*, and *FASN* genes; *DEAF1*, *KMT2D*, *KDELC1*, and *HSPBAD1* genes; and *DGK*, *LRRC7,* and *HECTD4* genes had a high probability of co-occurring (Figure 4). In the group of patients not previously exposed to radiation, the same was observed for mutations in *FAM13B*, *SPDYE3*, *PTEN*, *RYR2*, and *ACADVL* genes; *ABCC6*, *EPHB4*, *ADAMTS2*, and *POT1* genes; and *ZNF430* and *SNRNP200* genes. Importantly, similar to the overall analysis, mutations in the *PLCG1* and *KDR* genes were found to be mutually exclusive (Figure 4). This is interesting, as both proteins have been reported to be essential for blood vessel development, and *PLCG1* is a downstream signaling effector of *KDR* [22,27].

**Figure 5:**
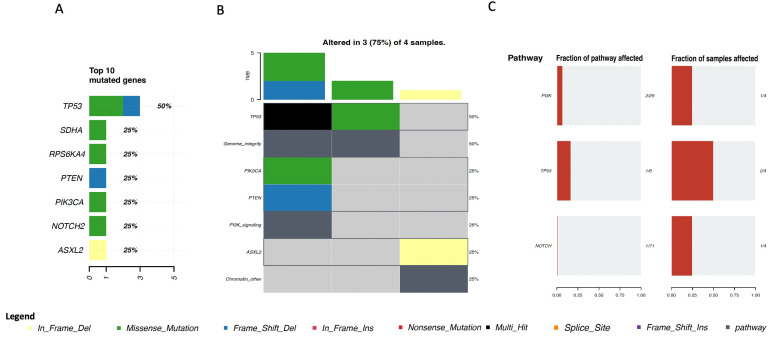
Genomic profile of other types of sarcomas of the breast. (**A**) Top 10 mutated genes in cohort A; (**B**) Oncoplot for cohort A. Each row represents one gene, and each column represents one sample; (**A**) Top 10 mutated genes in cohort C2; (**B**) Oncoplot for cohort C.2; (**C**) Enriched oncogenic signaling pathways in cohort C.2.

Available data from other breast sarcomas were also assessed, including two undifferentiated pleomorphic sarcomas/malignant fibrous histiocytomas/high-grade spindle cell sarcomas, one leiomyosarcoma, and one round cell sarcoma. Of note, these samples were all from the MSKCC cohort, and the sample population was small. Considering these limitations, *TP53*, *SDHA*, *RPS6KA4*, *PTEN*, *PIK3CA*, *NOTCH2*, and *ASXL2* were found to be the most mutated genes (Figure 5A), being mainly involved in genome integrity pathways, the *PI3K* signaling pathway, and chromatin remodeling (Figure 5B,C).

Moreover, clinical factors, such as tumor location, can have an impact on the mutational profile of the tumor. To investigate whether the mutational profile of the sarcoma changes with its location, a clinical enrichment analysis was conducted using the R package *“Maftools”*. This tool performs multiple groupwise and pairwise comparisons to identify enriched mutations for each category within a clinical feature, in this case, within different tumor locations. 

Clinical enrichment was performed for each group (angiosarcomas and other sarcomas). The *p*-value was set below 0.05.

**Figure 6:**
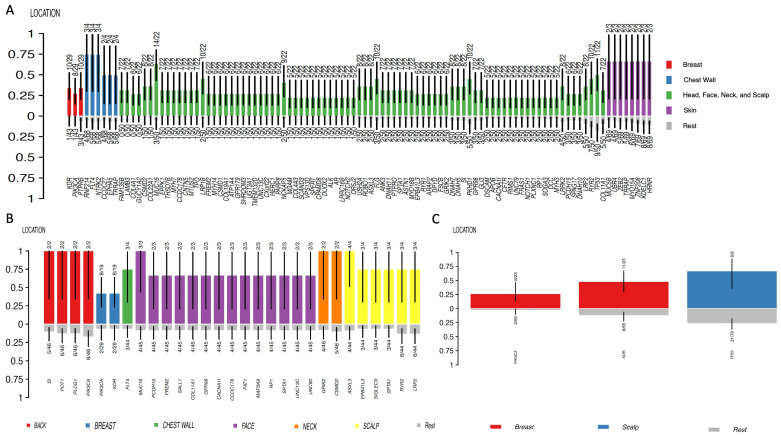
Clinical enrichment (CE) analysis according to angiosarcoma location. (**A**) CE for cohort A; (**B**) CE for cohort B; (**C**) CE for cohort C.1.

In the group of angiosarcomas, genes with enriched mutations were *KDR*, *PIK3CA*, and *PTPRB* in breast angiosarcoma; *TP53*, *ASXL3*, *EPB41L3*, *SIGLEC9*, *SPTA1*, *RYR2*, and *LRP2* in scalp angiosarcoma; *RNF214*, *FLT4*, *NTRK2*, *CCDC27*, *EPHA5*, and *TRRAP* in chest wall angiosarcoma; and *SI*, *POT1*, *PLCG1*, and *PIK3CA* in dorsal/lumbar angiosarcoma (Figure 6). *PIK3CA* was the only gene with mutations in angiosarcomas located in different topographies (Figure 6).

**Figure 7:**
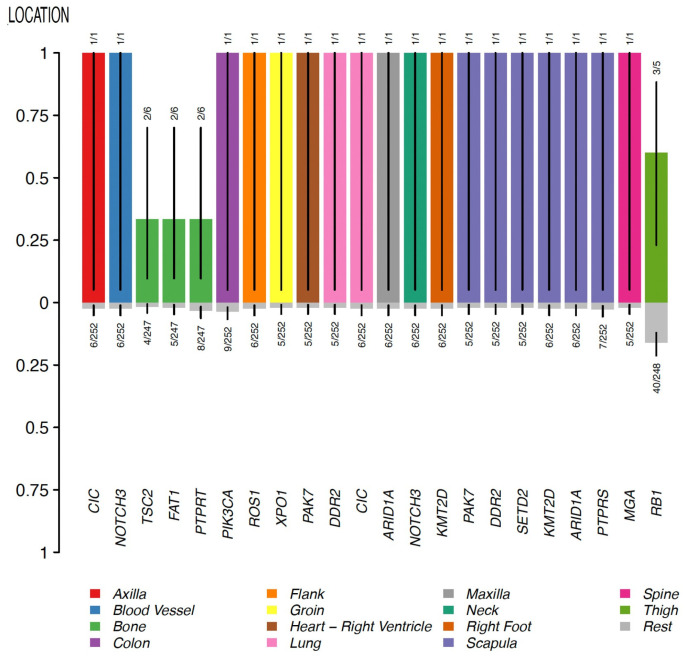
Clinical enrichment (CE) analysis for other types of sarcoma. CE for cohort C.2.

In the group of other sarcomas (which included undifferentiated pleomorphic sarcoma/malignant fibrous histiocytoma/high-grade spindle cell sarcoma, leiomyosarcoma, and round cell sarcoma), no enriched gene mutations were identified in breast topography. Conversely, tumors located in bone were found to have enriched mutations in *TSC2*, *FAT1,* and *PTPRT* genes; angiosarcomas of the colon were found to have enriched mutations in the *PIK3CA* gene; and angiosarcomas of the scapula were found to have enriched mutations in *PTPRS*, *ARID1A*, *KMT2D*, *SETD2*, *DDR2*, and *PAK7* genes (Figure 7).

These observations suggest that the mutational profile shifts according to the type and location of the sarcoma. The only significant exception to this rule was the *PIK3CA* gene, whose mutations appeared to be associated with sarcomas of different types and in various topographies. Although further studies are needed to validate this, this observation suggests an important role for the *PIK3CA* gene across different sarcoma subtypes.

Despite the potential functional relevance of these associations, the small sample size is a limitation of this analysis that should be taken into account when interpreting the results obtained.

## 5. Clinical Characteristics

Breast sarcomas, either primary or secondary, are rare entities, and consequently, there is a paucity of literature on the subject, especially studies describing the series of clinical cases. 

Although they are a heterogeneous group of non-epithelial tumors arising from the mesenchymal tissue of the breast with different natural histories, treatments, and prognoses, the clinical presentation is mostly not different from the most common breast cancer types. 

Most breast sarcomas present as unilateral, painless, firm nodules or lumps in the breast [2]. Their main distinguishing feature is size, as they tend to be larger, with a median tumor diameter of approximately 5 cm [2]. Some types of sarcoma, such as undifferentiated pleomorphic sarcoma, may have a fungating aspect and be profusely hemorrhagic in addition to being large [30]. 

Nodal involvement is typically rare [31], except in breast carcinosarcoma [32]. This is a rare subtype of metaplastic breast cancer in which the epithelial component may be an invasive ductal carcinoma, squamous cell carcinoma, lipid-rich carcinoma, or adenocarcinoma, and the mesenchymal component may contain different elements of fibrosarcoma, chondrosarcoma, osteosarcoma, liposarcoma, or leiomyosarcoma. Other types of sarcoma with potential lymphatic spread include angiosarcoma, rhabdomyosarcoma, clear cell sarcoma, synovial sarcoma, and epithelioid sarcoma [33].

A particular subtype of breast sarcoma—undifferentiated pleomorphic sarcoma—may present with a fever of unknown origin or neoplastic fever, as documented in a recent article in which neoplastic fever was accompanied by a rapidly growing mass in the breast that evolved into a swollen, violaceous, fame-like erythematous breast with thinned skin and nipple necrosis over a few months [34].

Another article describes six cases of breast sarcoma [35]. The median age at diagnosis was 59 years; all patients had localized disease without lymph node invasion or distant metastases, and none presented with multiple or bilateral lesions [35]. The article highlighted the lack of differences in the clinical presentation of patients’ different sarcoma subtypes (leiomyosarcoma, pleomorphic undifferentiated sarcoma, periductal stromal sarcoma, myxofibrosarcoma, and primary breast osteosarcoma) [35]. 

A large number of individual cases and a single meta-analysis of breast angiosarcoma have helped to paint a portrait of the clinical features of these tumors. Breast angiosarcoma represents less than 1% of all STS but is the most common histologic subtype of secondary breast sarcoma [36]. The literature on breast angiosarcoma highlights several possible clinical presentations. Evidence is derived from:

- The first published case of a collision tumor with concomitant and adjacent primary angiosarcoma and invasive carcinoma of the breast was in a 45-year-old patient [37]; this tumor presented as a lump in the breast [37]. Collision tumors are a rare entity, particularly in the breast.
- A rare case of breast angiosarcoma in a 17-year-old girl with an unusual clinical presentation [38]; this breast angiosarcoma initially mimicked an inflammatory breast carcinoma and then grew quickly as a mass, occupying the entire right breast with indistinct borders and poor mobility [38]. In addition, two erythematous patches on the skin and a cystic lesion of approximately 3 by 4 cm surrounded by indurated tissue underneath the larger erythematous patch were also observed [38].
- A review of twenty-two cases of primary and secondary breast angiosarcomas and an additional meta-analysis from Roswell Park Comprehensive Cancer Center described primary breast angiosarcomas (i.e., those that develop de novo, without prior breast radiation exposure) as typically occurring in 30-to-50 year-old women and presenting as a large mass without skin changes [36]. On the other hand, secondary breast angiosarcomas (typically associated with radiation exposure to the breast and/or chest wall or with chronic lymphedema following breast surgery and lymph node dissection) are typically reported in 60–70-year-old women and present as ecchymosis with or without ulceration [36].
- Radiation-induced breast angiosarcoma may also present as skin discoloration or purplish-red nodules, as thickening or elevation of the skin without color change, and/or as diffuse skin extension of lesions of various morphologies [31]. Another common feature is the presence of multifocality with microsatellite lesions [31].

In general, the risk for all radiation-induced breast sarcomas peaks at 10 years and, although declining, remains high for more than 20 years [31]. 

In summary, breast sarcomas usually present as unilateral, painless, rapidly growing breast masses. Changes in skin color and texture may occur in the most aggressive sarcoma subtypes. Nodal involvement is rare, except in certain subtypes.

## 6. Diagnosis and Staging

Breast sarcomas are a particularly heterogeneous group of mesenchymal neoplasms. Although there is no consensus on the inclusion of phyllodes tumors (epithelial component), carcinosarcomas (epithelial origin), and desmoid tumors of the breast under this designation [4,39,40], specific aspects of phyllodes and desmoid tumor staging will also be discussed in this section.

Diagnostic mammography and breast ultrasound are not the best methods for identifying and characterizing breast sarcomas [2]. Mammography findings can be unspecific, as breast sarcomas may present as a nonspiculated dense mass without microcalcifications and with indistinct margins, which are findings that may resemble benign fibroadenomas [41]. Furthermore, mammography may appear normal in the presence of a suspicious sarcomatous lesion [41]. Although breast ultrasound may provide additional clues, it does not show specific changes, and most sarcomatous lesions are hyperechoic without shadows [42]. 

MRI with contrast is the imaging modality of choice because it allows the identification of suspicious malignant changes (irregularly bordered oval masses with T2 hyperintensity and heterogeneous rapid contrast enhancement and washout) and a proper assessment of the local extension of the disease (showing the extent of involvement of the surrounding skin, fascia, and muscular structures), which are crucial for planning the surgical and radiotherapy approaches [2,41,42,43,44,45]. However, it should be emphasized that MRI may underestimate skin involvement, especially in cases of cutaneous angiosarcoma [2]. 

The diagnosis of breast sarcoma cannot be based on imaging alone. A biopsy is mandatory and should be performed when there is a high index of suspicion based on clinical observation or imaging assessment. The use of fine-needle aspiration is not recommended because histologic subtype and grade cannot be accurately determined from fine-needle aspiration specimens, and therefore the technique is associated with low diagnostic accuracy and false-negative results [2,39,43,46,47]. A core needle biopsy should be the initial approach of choice, but if inconclusive, an incisional or excisional biopsy (depending on the lesion size) should be performed [2]. Cutaneous radiation-associated angiosarcoma can be diagnosed with a punch biopsy [2]. 

Pathologic review should be performed by expert sarcoma pathology teams to achieve an accurate diagnosis and precise histologic subtype determination. In terms of morphologic and immunohistochemical features, breast sarcomas share the main characteristics of STS [43]. Immunochemistry is also crucial for a proper differential diagnosis with other tumors and the exclusion of epithelial origin/component.

Benign and borderline phyllodes and desmoid tumors typically do not require additional radiographic studies for staging purposes, as they have little to no distant metastatic potential [2]. Patients diagnosed with any breast sarcoma should undergo a computed tomography (CT) scan of the chest, as the lungs are the most common site of metastases globally [2]. A CT scan of the abdomen and pelvis and a bone scan should be considered in patients with breast angiosarcoma, given its propensity for secondary spread to the liver and bone [2]. In addition to angiosarcoma, a CT scan of the abdomen and pelvis is also recommended for breast myxoid/round cell liposarcoma, epithelioid sarcoma, and leiomyosarcoma [48]. Positron emission tomography (PET) scans are another alternative, but their role remains unclear. 

Breast sarcoma staging should follow the TNM AJCC UICC 8th edition system for STS of the extremities and trunk, which considers primary tumor characteristics, regional lymph node involvement, presence of distant metastases, and histologic grade (based on tumor differentiation, mitotic count, and degree of necrosis) [49]. Primary tumor staging is based solely on tumor size [2]. As noted above, and unlike breast carcinoma, breast sarcoma rarely metastasizes to regional or distant lymph nodes, and the presence of regional lymph node involvement is considered stage IV disease [2]. 

Phyllodes tumors are classified by the World Health Organization as benign, borderline, or malignant (with varying prognosis and management), and their classification is based on the degree of stromal cellular atypia, mitotic activity, tumor margin characteristics (well-defined vs. infiltrative), and the presence of stromal overgrowth [2]. Desmoid tumors are not classified according to the AJCC UICC STS classification system because they do not show a propensity for regional nodal or distant spread [2]. To date, there is no consensus on the most appropriate staging system for desmoid tumors [2]. 

## 7. Prognostic and Predictive Factors

Breast sarcomas have been retrospectively associated with clinicopathologic prognostic and predictive factors.

### 7.1. Prognostic Factors

An analysis of the SEER database by Osman et al. showed that secondary breast sarcoma has a worse prognosis compared to primary breast sarcoma, behaves more aggressively, and has poorer outcomes, with a median overall survival of about 3 years [49]. The same analysis showed that angiosarcoma is the most common subtype of breast sarcoma and is typically associated with a worse prognosis [49]. Surprisingly, other analyses revealed that its prognosis does not seem to be associated with histologic grade [2]. Angiosarcomas account for a much higher proportion of secondary breast sarcomas [2] and are also more prevalent in older ages as secondary than as primary sarcomas [50,51]. In their analysis, Yin et al. showed that osteosarcoma and fibrosarcoma/liposarcoma were associated with the worst and best prognosis among angiosarcomas, respectively [52]. 

In addition to the histologic subtype, other prognostic factors have been identified in breast sarcoma. In the same analysis, Yin et al. showed that older age and regional and distant tumor spread were associated with an increased risk of death in primary breast sarcoma, while a higher histologic grade, larger tumors, positive margins, and a mastectomy (vs. breast-conserving surgery) were associated with worse outcomes in breast sarcoma [52].

### 7.2. Predictive Factors

Unlike epithelial breast carcinoma, whose intrinsic subtypes have specific predictive biomarkers (e.g., hormone receptors or human epidermal growth factor receptor 2), the classification and management strategies for breast sarcoma are not as consistently based on predictive biomarkers and, similar to other STS located in different body topographies, generally follow a “fit-for-all principle”. 

Yin and colleagues found that adjuvant radiotherapy seemed to improve survival only in tumors larger than 5 cm [52]. 

A recent analysis of the National Cancer Database performed by Lee et al. showed that surgical excision was the treatment modality with better outcomes regarding overall survival [33]. Adjuvant radiation showed more efficacy in larger tumors and those with positive margin resections [33]. Regarding adjuvant systemic therapy, patients who received chemotherapy were associated with worse outcomes in this analysis, but the authors noted that this treatment was usually chosen for high-risk diseases [33]. Finally, treatment at a designated cancer center or academic teaching hospital was associated with better overall survival [33].

## 8. Treatment

### 8.1. General Management

As a rare group of diseases, the management of breast sarcomas is mainly guided by small retrospective case or series reviews as well as extrapolation from evidence on STS of the extremities and chest wall. A multidisciplinary approach in a specialized center with experienced medical oncologists, surgeons, radiation oncologists, and pathologists is preferred, as recommended for the management of non-breast STS [53,54].

Overall, surgery is the only potentially curative treatment for these tumors. The optimal type and extent of surgery have not been definitively established and depend on tumor and breast size (as in breast carcinoma) and histology [43]. The goal of surgery is to achieve adequate surgical margins, as this is one of the most important prognostic factors and determinants of long-term survival [40,43,55]. 

Breast-conserving surgery is an option for breast sarcomas because, with the exception of angiosarcomas, they are rarely multicentric. This specific histology will be further discussed in this review. STS generally metastasize either by direct local invasion or by hematogenic route, and breast sarcomas follow this pattern. Metastases to regional lymph nodes occur in less than 5% of cases, and lymphadenectomy may not improve outcomes [56,57]. Patients with clinically suspicious axillary lymph nodes can be evaluated with an ultrasound-guided fine-needle biopsy, and lymphadenectomy is an appropriate option for those with proven lymph node involvement.

Adjuvant radiotherapy has not been shown to benefit these tumors in randomized trials, and retrospective data are conflicting [40,56,58,59,60,61,62]. For STS, re-excision is preferred in the setting of positive or close surgical margins, and this approach can also be used for breast sarcomas [53]. Radiotherapy may be used when re-excision is not feasible or may lead to significant morbidity or unfavorable cosmetic outcomes. Radiotherapy may also be considered for large (>5 cm) and high-grade tumors with close (deep) margins, but it should not be used to compensate for inadequate surgery. The management of radiation-associated sarcomas (mainly angiosarcomas in this case) is discussed below.

Adjuvant chemotherapy is not universally recommended and, similar to non-breast STS, should be discussed on an individual basis. There are no randomized trials of adjuvant therapy in this specific setting, and retrospective data are generally pooled from larger studies, including many types and locations of STS.

The management of patients with metastatic breast sarcoma is similar to that of patients with metastatic STS of other topographies, with doxorubicin-based regimens (with doxorubicin alone or in combination with ifosfamide) being the first-line choice, except in patients with treatment-related sarcomas who have been previously treated with doxorubicin and who have a significant risk of cardiotoxicity with higher cumulative doses [2,53]. According to international guidelines for the treatment of metastatic STS, various systemic regimens can be used (including chemotherapy agents or tyrosine kinase inhibitors [TKIs]), some of which are histology-specific [53]. The use of immune checkpoint inhibitors for the treatment of STS is not standard, but preliminary evidence suggests their usefulness in the treatment of specific sarcomas, namely angiosarcomas [2]. Finally, patients with isolated or limited lung metastases may be considered for a wedge resection of these lesions, which may provide 5-year overall survival rates as high as 40% [2]. 

### 8.2. Angiosarcoma

Breast angiosarcoma is a highly aggressive malignancy whose optimal management is based on expert opinion (due to its rarity) and should be discussed in a multidisciplinary context [31]. 

Early disease can be managed with surgery, radiotherapy, or chemotherapy, while radical surgery with R0 resection remains the cornerstone of treatment for localized breast angiosarcoma and should be performed in experienced, high-volume centers [31]. Positive surgical margins have been consistently reported to have a negative impact on overall survival and an increased risk of local recurrence [63].

The best surgical approach is uncertain due to the lack of long-term outcome data. However, prior radiation-induced tissue changes and the diffuse infiltrative margins of angiosarcoma make complete tumor excision a surgical challenge. Radical mastectomy more often achieves negative margins, and wide local excision should only be an option for low-grade tumors up to 5 cm in diameter [64].

Breast angiosarcoma usually metastasizes by hematogenous spread and rarely to regional lymph nodes. Therefore, sentinel lymph node biopsy or systematic lymph node dissection are not recommended [31,65]. 

A limited number of studies have shown the significant clinical benefit of adjuvant radiotherapy following radical surgery. This includes a trend toward improved recurrence-free survival and overall survival [31,36]. Neoadjuvant radiotherapy is not currently recommended because adjuvant radiotherapy provides better local control [66]. Finally, improved prognosis has been demonstrated with adjuvant radiotherapy in two studies that included patients with both primary and radiotherapy-induced breast angiosarcoma [39,66]. 

Similar to other STS, the role of adjuvant chemotherapy in breast angiosarcoma remains uncertain. The general theory is that adjuvant chemotherapy has a significant benefit in reducing the risk of local recurrence. Evidence of a survival benefit was reported in a small study in patients with secondary breast angiosarcoma but not in those with primary angiosarcoma [31]. Another small study including primary and secondary breast angiosarcomas reported a trend toward improved recurrence-free survival with adjuvant chemotherapy, which was not confirmed in a larger meta-analysis. Finally, a nationwide U.S. analysis of breast angiosarcoma showed that chemotherapy after surgery was associated with prolonged overall survival in patients with large tumors (>5 cm) [36,67].

Data on neoadjuvant chemotherapy are even scarcer. It may be an option for locally advanced, inoperable disease, seeking to downsize the tumor and thus facilitate R0 resection [31].

Due to the aggressive nature of angiosarcoma, approximately 50% of patients with localized disease develop local recurrence and distant metastases [68].

Cytotoxic chemotherapy is the standard first-line treatment for advanced disease. The choice of chemotherapy should take into account the patient’s comorbidities and the risk of drug-related toxicities [69]. Anthracyclines are commonly used, particularly doxorubicin, either as monotherapy or in combination with ifosfamide. Doxorubicin is the mainstay of treatment for metastatic STS [70]. Sher et al. reported an overall response rate to first-line anthracycline–ifosfamide combination therapy of 48% in 29 patients with metastatic breast angiosarcoma [71]. Liposomal pegylated doxorubicin showed similar results in the treatment of breast angiosarcoma, with a favorable toxicity profile. Overall, liposomal pegylated doxorubicin is an effective treatment option for patients who cannot tolerate doxorubicin toxicity. Taxanes may also be used, with paclitaxel considered an active monotherapy in angiosarcoma and often used in the first- or second-line setting of metastatic disease [69,72]. In ANGIOTAX, a small phase II trial of 30 angiosarcoma patients (33% of whom had breast angiosarcoma), progression-free survival (PFS) at 4 months was 45% [72]. 

Although the optimal sequence of anthracycline- and taxane-based chemotherapy remains controversial, both are considered active and recommended in the treatment of angiosarcoma [73]. Gemcitabine and trabectedin may also be considered based on extrapolated evidence from STS trials [31]. 

In addition to cytotoxic chemotherapy, TKIs may be used to treat advanced breast angiosarcoma. TKIs have been used as targeted therapy for angiosarcoma through the inhibition of the vascular endothelial growth factor (VEGF)/vascular endothelial growth factor receptor (VEGFR) signaling pathways.

Pazopanib is a multi-target TKI against VEGFR1, VEGFR2, VEGFR3, and platelet-derived growth factor receptors (PDGFR) that is approved for the treatment of metastatic STS after anthracycline-based chemotherapy [74,75]. A retrospective EORTC study of pazopanib in advanced vascular sarcomas showed an ORR of 20% in 40 angiosarcoma patients, with no differences between radiation-induced and non-radiation-induced angiosarcomas [76].

Sorafenib is another multikinase agent targeting RAF, PDGF, VEGFR2, VEGFR3, and c-Kit that has shown activity against angiosarcoma in a phase 2 trial in STS [77,78]. 

### 8.3. Other Breast Sarcomas

In addition to breast angiosarcoma, breast liposarcoma, leiomyosarcoma, rhabdomyosarcoma, and chondrosarcoma also have management specificities. 

Breast liposarcoma accounts for 0.3% of all breast sarcomas and most commonly affects people between the ages of 45 and 55 [79]. It typically develops from pre-existing benign breast lesions, such as lipoma, fibroadenoma, and phyllodes tumor [80]. Breast liposarcoma typically occurs as pure primary liposarcoma or cystosarcoma phyllodes, is more often unilateral, infiltrative, and either well-circumscribed or multinodular, and more often presents as a painful or painless soft, slowly growing mass (the growth rate and invasiveness depend on the degree of differentiation) [80]. 

The mainstay of treatment for breast liposarcoma is complete surgical resection with tumor-free margins (R0), although the debate over the use of breast-conserving surgery versus mastectomy is ongoing [80]. Better survival outcomes are apparently seen with breast-conserving surgery compared to mastectomy, but the number of studies is particularly sparse due to the rarity of this entity [80]. The benefit of axillary node dissection is controversial, with some evidence suggesting no benefit as the axilla is not a region of frequent metastization [80,81]. 

The benefit of radiotherapy and chemotherapy in localized primary breast liposarcoma is unclear, although adjuvant chemotherapy (regimens and agents known to be more effective in the treatment of liposarcoma) and adjuvant radiation therapy may be recommended for high-risk patients (non-negative surgical margins, higher-grade liposarcomas, or size >5 cm), similar to what happens for liposarcomas in other regions. Due to the rarity of breast liposarcoma, there are no specific guidelines for the surgical management and subsequent therapeutic approach of these tumors. In cases of disseminated breast liposarcoma, the use of chemotherapy and radiotherapy should follow what is advocated in international guidelines for metastatic STS. 

Breast leiomyosarcoma is also rare, with only about 60 cases reported in the literature to date [81]. It represents 2.5–6% of all primary breast sarcomas [81]. Breast leiomyosarcoma is more common in postmenopausal women, although a number of reports describe cases in younger patients [81]. A recent systematic review reported a median age of 56.1 years and an age range of 18–80 years in these patients [81]. Breast leiomyosarcoma is believed to arise from the smooth muscle cells of the lactiferous ducts or blood vessels or from the erector pili muscle at the periphery of the areola [81,82] and presents as a large, painless, and firm mass within the breast [81]. 

As with other breast sarcomas, the only curative approach for breast leiomyosarcoma is surgery, with radical mastectomy being the standard procedure [82]. Adequate surgical margins appear to be of paramount importance, with a 3-cm margin considered appropriate by Fujita et al. However, achieving such margins may not be feasible for all cases [81,82,83]. Axillary lymph node evaluation is not widely recommended unless the preoperative diagnosis of leiomyosarcoma is uncertain [81], and axillary lymph node dissection is also not generally recommended given the low risk of regional lymphatic spread; the fact remains that these neoplasms are more likely to spread by hematogenous route to the lung, bone, liver, and central nervous system, and that the vast majority of cases of axillary adenopathy associated with breast leiomyosarcoma correspond to hyperplasia rather than metastases [82]. 

Ilyas et al. performed a systematic review of the 54 cases of breast leiomyosarcoma reported in the literature up to 2019. They showed that simple mastectomy with or without axillary lymph node biopsy or lymphadenectomy was performed in most patients (51.9%; n = 28), wide local excision in 27.8% (n = 15), and radical or modified radical mastectomy in 20.3% (n = 11) [81]. Interestingly, axillary lymph node evaluation was performed in a relevant number of patients (36.3%, n = 20) as part of mastectomy or with wide local excision and was not positive for malignancy in any of them [81]. Regarding recurrence patterns, local recurrence was observed in seven patients (12.7%), six of whom had recurrence between 18 months and 4 years after a wide local excision with resection margins ranging from 5 mm to 2 cm [81]. Local recurrence after mastectomy has only been reported in one case of locally advanced breast leiomyosarcoma involving the pectoral muscle [81]. 

The role of radiotherapy in the management of localized breast leiomyosarcoma is better established in the adjuvant setting. Local control of recurrence and disease-free survival may be improved by the use of adjuvant radiotherapy following breast-conserving resection, particularly in the absence of negative resection margins [82]. Due to the significant risk of recurrence, adjuvant radiotherapy should be delivered at a tumoricidal dose to the entire breast and at least 60 Gy to the tumor bed after positive margin resection [82]. Adjuvant radiotherapy is also recommended for high-grade breast leiomyosarcomas and for breast leiomyosarcomas larger than 5 cm, regardless of resection margins [82]. 

The benefit of adjuvant chemotherapy in breast leiomyosarcoma is less clear and cannot be considered a standard of care [82]. It should be guided by validated risk prediction tools such as SARCULATOR and PERSARC, with high-grade and large (>5 cm) tumors typically benefiting the most [82]. Treatment regimens should include doxorubicin and dacarbazine, as these are the agents known to be more effective in leiomyosarcoma [82]. 

In the systematic review by Ilyas and colleagues, nine of the 54 patients with breast leiomyosarcoma had received either adjuvant chemotherapy or adjuvant radiotherapy [83]. In another study, 27 systemic recurrences were reported in seven patients (12.7%), occurring in the liver, lung, brain, bone, and contralateral breast [81].

The use of chemotherapy and radiotherapy in metastatic breast leiomyosarcoma should follow the international guidelines for metastatic STS. Pazopanib is another treatment option beyond chemotherapy, namely in patients with metastatic non-adipose STS after prior chemotherapy [82]. 

Embryonal and alveolar rhabdomyosarcomas (ERMS and ARMS, respectively) are also very rare sarcomas. ERMS most commonly occurs in the head and neck and in the genitourinary tract in children aged between 1 and 14 years, while ARMS most commonly develops in the head and neck in children and in the extremities and trunk in adults, being more prevalent in older children, adolescents, and young adults [84,85]. To date, only approximately 26 cases of breast ARMS have been reported in the literature, and previous studies led by the Intergroup Rhabdomyosarcoma Study Group reported that primary breast ARMS accounted for only 0.17% of all rhabdomyosarcoma cases between 1972 and 1992 [84,85,86]. Breast rhabdomyosarcoma most commonly affects children and young adults and often presents as a breast mass that may be initially misdiagnosed as a benign fibroadenoma, leading to significant diagnostic delay [85]. 

Breast rhabdomyosarcomas are typically aggressive sarcomas with low survival and high metastatic rates. Breast ARMS typically spreads to the lungs, bones, and brain, but vaginal, orbital, and colonic metastases have also been reported [84]. The mainstay of the treatment is a prompt and complete surgical mastectomy followed by a breast reconstruction [84]. 

Primary breast chondrosarcomas are even rarer breast sarcomas, with only a few cases described in the literature [87]. Mastectomy is the preferred surgical approach when the tumor involves all four quadrants, whereas quadrantectomy may be recommended for tumors involving only one quadrant or of small size [87]. Lymph nodes are rarely involved in breast chondrosarcomas; therefore, axillary lymphadenectomy should not be combined with mastectomy as a standard procedure [87]. These tumors are not typically chemo- or radiosensitive [87]. 

## 9. Specificities and Management of Breast Phyllodes and Desmoid Tumors

### 9.1. Phyllodes Tumors of the Breast

Phyllodes tumors of the breast are a rare fibroepithelial neoplasm, accounting for 0.3% to 1% of all breast tumors [88,89]. They are typically large and rapidly growing and primarily occur in middle-aged women (40–50 years of age), about 15 to 20 years later than fibroadenomas, which are the main differential diagnosis to consider [88,90].

Genetic risk factors for phyllodes tumors are largely unknown, but the literature reports that patients with Li–Fraumeni syndrome, a rare inherited condition associated with an increased risk of a number of cancers, including breast cancer, have a higher risk of developing these tumors [88]. In addition, rare cases of phyllodes tumors in men are often associated with gynecomastia, suggesting a role in hormonal imbalance [88,91]. Researchers have stated that stromal induction of phyllodes tumors may occur due to growth factors produced by the breast epithelium. Trauma, pregnancy, increased estrogen activity, and lactation have occasionally been implicated as factors stimulating tumor growth. The nature of these factors is not well understood, but endothelin-1, a stimulator of breast fibroblast growth, may contribute [88,92]. Unlike breast carcinoma, phyllodes tumors originate outside the lobules and ducts, in the connective tissue of the breast, including in the ligaments and fatty tissue surrounding the lobules, ducts, lymph, and blood vessels of the breast [88,93]. These characteristics make the differential diagnosis between these tumors and fibroadenomas (both fibroepithelial tumors) challenging. The difficulty in the differential diagnosis is probably related to these tumors’ common origin, which was uncovered by the identification of a common somatic mutation—MDM12—by genomic sequencing. Given that fibroadenomas have a low risk of local recurrence, while phyllodes tumors have a high risk and potential for metastases, an accurate diagnosis is crucial for selecting the right therapeutic approach. It is also important to distinguish between the three histologic subtypes of phyllodes tumors. Although it is acknowledged that most phyllodes tumors are benign, their diagnosis must be made with caution, as approximately 22% present with metastases, with lung and bone being the most common metastatic sites [94]. This emphasizes the need for investigation to improve the molecular classification of these tumors. Although no chromosomal aberrations specific to phyllodes tumors have been described to date, some authors have reported that low-grade and high-grade (borderline/malignant) tumors segregate into two genetic groups based on genomic alterations, with high-grade tumors consistently showing 1q gain and 13q loss and low-grade tumors showing few or no alterations [95]. Immunohistochemistry studies are also ongoing, namely on potential biomarkers, with p53 expression and Ki67 index reported in some studies as significantly associated with disease-free and overall survival but showing no association with recurrence or clinical behavior in others [96]. PAX3 and SIX1 immunohistochemistry and gene expression have recently been identified in borderline and malignant phyllodes tumors and correlated with poor clinical outcomes [97].

The primary treatment for phyllodes tumors is surgery, but the optimal extent of this treatment (whether breast-conserving surgery or mastectomy) has been a matter of debate in recent years. Historically, mastectomy has been the treatment of choice, especially for patients with borderline or malignant phyllodes. Today, the generally accepted approach for patients with all histologic types of phyllodes tumors is breast-conserving surgery with a clear margin of healthy tissue, and mastectomy is warranted only when surgery cannot achieve an adequate tumor-free margin or satisfactory cosmesis. Despite the higher incidence of local recurrence following breast-conserving surgery, studies have shown no differences between this approach and mastectomy regarding metastasis-free or overall survival. Because phyllodes tumor metastases to the axillary lymph nodes are very rare, there is no indication for sentinel lymph node biopsy or elective axillary lymphadenopathy [89,93]. Surgical margins and the subsequent risk of local recurrence according to histologic subtype are another matter of debate in the surgical approach for phyllodes tumors. Undoubtedly, the presence of tumor cells in the resection margin is a strong prognostic factor for local recurrence, especially in cases of borderline and malignant tumors. In a large study of 605 patients with phyllodes tumors conducted by Tan et al., surgical margin status (positive vs. negative) appeared to be an independent predictive factor for recurrence-free survival. Spitaleri et al. reported a mean local recurrence rate of 31.5% in patients with positive surgical margins. According to Strode and colleagues, the appropriate treatment for malignant phyllodes tumors is surgical excision with negative margins. Clear margins have thus been identified as a key factor for low local recurrence in these tumors. However, the appropriate width of tumor-free margins remains controversial. NCCN and several authors recommend a free margin of 1 cm, but often it is difficult to achieve both a positive cosmetic outcome and a 1 cm margin. A growing number of authors suggest that a margin of at least 1 mm (tumor-free margin) may be sufficient to prevent local recurrence, especially in patients with benign or borderline phyllodes tumors. Regarding borderline and malignant phyllodes, a margin of at least 1 mm all around the tumor is sufficient, and the previously considered 1 cm-wide margin is not required. In contrast to benign phyllodes tumors, a negative margin is likely to be insufficient in borderline and malignant tumors, suggesting that surgical revision of the margin may be necessary in patients with a margin <1 mm. Therefore, the ideal margin width for these patients remains to be determined. Patients with borderline and malignant phyllodes tumors should be closely followed [93].

Regarding subsequent treatment, there is no well-established adjuvant therapy for high-grade phyllodes, in part due to the controversial roles of adjuvant radiotherapy and chemotherapy and the lack of sufficient prospective data. Adjuvant radiotherapy has been more frequently used recently. Data from a large retrospective study suggested that radiotherapy could prolong the time to local recurrence and decrease the local recurrence rate without significantly affecting survival. However, there is a paucity of data regarding the relationship between radiotherapy and metastases. Results of a subgroup analysis suggested that radiation may be more effective in younger patients (<45 years), patients with larger or malignant tumors, and patients with larger excisions. A meta-regression analysis also confirmed the importance of margin status in local control. However, the type of surgery showed less impact on disease control, suggesting that for phyllodes tumors with high malignancy, radiotherapy should be used as adjuvant therapy regardless of the surgery type [90]. Regarding systemic treatment, although the epithelial component of most phyllodes tumors contains estrogen receptors (58%) and/or progesterone receptors (75%), endocrine therapy has no proven role in the treatment of these tumors. Similarly, there is no evidence that adjuvant cytotoxic chemotherapy reduces recurrence or death. For the rare patients who experience systemic recurrence, treatment should be the same as that recommended for other STS [98].

Many questions persist about these rare cancers. Accurate preoperative pathologic diagnosis allows adequate surgical planning and avoids reoperation. The distinction between phyllodes tumors and fibroadenomas is important but can be difficult on a core biopsy. Prospective studies investigating the efficacy of adjuvant therapy are also required, but the fact that most published data are retrospective precludes comparisons between available treatments. The management of phyllodes tumors requires a multidisciplinary approach with input from a surgical oncologist, an oncologist, a pathologist, and a radiologist, along with oncology nurses, all working together as an interprofessional team to achieve the best clinical outcomes.

### 9.2. Breast Desmoid Tumors

Desmoid tumors, also known as aggressive fibromatosis, are rare mesenchymal tumors. They account for 0.03% of all tumors and have an estimated incidence of 2 to 4 per million people per year [99]. Desmoid tumors are more common in people aged between 15 and 60 years (with a peak incidence between 30 and 40) and have a female:male ratio of 2 to 3:1 [99]. These tumors result from the monoclonal proliferation of myofibroblasts in the musculoaponeurotic structures that connect, support, and surround various body segments and organs [99]. The most common sites include the mesentery, abdominal and chest walls, and extremities [100]. Breast desmoid tumors represent 4% of all extra-abdominal desmoid tumors and 0.2% of all breast neoplasms [100]. 

Some cases of desmoid tumors seem to occur in association with pregnancy and the use of estrogen-containing oral contraceptives [101]. Accordingly, they usually stabilize or regress in postpartum and menopause, in accordance with the hormonal variations that seem to be implicated in the evolution of these tumors [101]. Additional risk factors include prior trauma and surgery (in fact, up to 44% of patients with breast desmoid tumors have had prior breast surgery [2]), although these tumors are not conceptually considered “treatment-related” [101]. 

Around 85–90% of desmoid tumors are sporadic, associated with mutations in the CTNNB1 gene, which encodes β-catenin, and 5–10% develop in the context of familial adenomatous polyposis (FAP), in which a germline mutation of the adenomatous polyposis coli (APC) gene is detected [2,101]. CTNNB1 and APC mutations are believed to be mutually exclusive [97]. Biologically, desmoid tumors may be driven by constitutive activation of the Wnt/APC/β-catenin pathway (resulting from either activating mutations in the β-catenin oncogene CTNNB1 or inactivating mutations in the tumor suppressor gene APC) and activation of the Notch pathway (resulting from crosstalk between the dysregulated Wnt and the Notch pathways) [101]. Although the Wnt pathway is difficult to target therapeutically without disrupting the system of normal somatic stem cell function in cellular repair and tissue homeostasis, the dysregulation and activation of the Notch pathway offer interesting potential therapeutic targets [101]. For example, γ-secretase plays a critical role in Notch signaling by cleaving the intracellular domain of Notch (with subsequent translocation to the nucleus to activate gene transcription), and γ-secretase inhibitors (GSI) have now emerged as a potential compelling treatment for desmoid tumors [101]. 

Desmoid tumors are typically locally aggressive growing tumors that do not metastasize and exhibit a clinical behavior that is different from classical STS [100]. Although this is the expected behavioral pattern, their natural history varies, ranging from an asymptomatic, indolent course to aggressive invasion and infiltration of neurovascular structures and vital organs, potentially leading to pain, disfigurement, organ dysfunction, and rarely death [101]. 

Conceptually, the mainstay treatment for desmoid tumors is surgery, although the significant rates of local recurrence and poor functional outcomes following surgery have prompted a shift toward non-surgical strategies, such as active surveillance or medical management [101]. Spontaneous regression may occur in up to 10–20% of these tumors; thus, a period of surveillance is advocated for almost all asymptomatic patients, as it may lead to long-term avoidance of local and systemic treatments that are not without side effects [101]. Patients with symptomatic desmoid tumors, desmoid tumors that progress during surveillance, and desmoid tumors located in specific anatomic topographies where progression could be detrimental should be actively treated [101]. 

A wide variety of locoregional approaches are available, ranging from cryoablation to radiotherapy and high-intensity focused ultrasound [101]. 

A number of systemic treatment approaches have been explored, including hormonal therapy and non-steroidal anti-inflammatory drugs (NSAIDs). Regarding the first, although estrogen has long been considered to modulate desmoid tumors, evidence concerning the effectiveness of anti-estrogen treatment in these tumors is restricted to case series and single-arm trials, and consequently, treatment guidelines do not advocate hormonal therapy for these tumors. Regarding NSAIDs, the overexpression of cyclooxygenase-2 (COX-2) in desmoid tumors has prompted research on the use of these agents (some in combination with hormonal therapy) as disease-modifying therapies in this context. However, no randomized, prospective trials have been conducted, only open-label and observational studies, and therefore guidelines recommend them only for pain relief [101]. 

Evidence for the treatment of desmoid tumors with chemotherapy comes from retrospective and prospective non-randomized studies [101]. The use of different chemotherapy regimens was investigated in a recent study; low-dose methotrexate plus vinblastine or vinorelbine or a conventional anthracycline-containing regimen achieved disease control rates of 64–100%; however, their use may be associated with significant hematologic toxicity [101]. TKIs targeting VEGFR and/or PDGFR inhibit the growth and progression of desmoid tumors, as shown in phase 3 randomized, double-blind, placebo-controlled trials, including one trial investigating the use of sorafenib that showed an impressive PFS rate of 81% [102,103]. Other prospective studies have specifically evaluated the benefit of imatinib [102,104] and pazopanib [102,105] in the treatment of progressive desmoid tumors and supported their use in these patients. However, the long-term safety and tolerability of these agents still need to be appropriately assessed [101]. The treatment of desmoid tumors with agents specifically targeting Wnt (as tegavivint and ipafricept) and Notch (as the gamma-secretase inhibitor [GSI] nirogacestat, AL102, and crenigacestat) pathways has recently come into the spotlight. GSI currently seems to be the most promising drug class, with nirogacestat being the most extensively studied agent in these tumors [101]. The randomized, double-blind, placebo-controlled phase 3 DeFi study of nirogacestat 150 mg twice daily in 142 adult patients with progressive desmoid tumors showed that the GSI was associated with a PFS (primary endpoint) benefit over placebo, as well as a higher likelihood of event-free disease at 2 years, higher objective response rates, a shorter median time to response, and higher complete response rates [106]. In addition, nirogacestat showed a favorable toxicity profile. Diarrhea (84%), nausea (54%), fatigue (51%), hypophosphatemia (42%), and maculopapular rash (32%) were the most frequent adverse events, but 95% were grade 1 or 2 [106]. AL102 is currently being evaluated in the phase 2/3 RINGSIDE trial [107]. This trial, which was initiated in March 2021 with 192 patients with progressive desmoid tumors, consists of two parts: part A, an open-label, dose-finding phase in which AL102 will be given at oral doses of 1.2 mg daily or 2–4 mg twice weekly; and part B, a double-blind, placebo-controlled phase designed to evaluate the optimal dose identified in part A [107].

The choice of the treatment modality and agent and of the therapy sequence will be mainly shaped by individual patient characteristics (such as age, comorbidities, and performance status), the nature and functional impact of desmoid tumor-associated symptoms, tumor characteristics (such as size, location, degree of invasiveness, and growth rate), the number and type of risk factors associated with tumor recurrence, treatment characteristics (such as expected response rate, ease of administration, and expected toxicity), and the degree to which each particular treatment modality incurs a response [101]. 

As mentioned above, for breast desmoid tumors, a primary nonsurgical approach with active patient monitoring seems a reasonable approach for patients with stable asymptomatic tumors [2,100]. Evidence from direct and indirect observations from several studies supports this active surveillance strategy, provided that core biopsy findings are unequivocal and that close follow-up by MRI is feasible. The surveillance strategy should include serial MRI assessments within 4 to 6 weeks of diagnosis, at 3–6-month intervals for the first 3 years, and then annually [5,102]. The series published by Duazo-Cassin et al. provided direct evidence supporting an active surveillance strategy by showing that 15 of 17 patients with breast desmoid tumors who underwent primary active monitoring did not require active treatment [100,108]. Another recent study evaluated the imaging, histopathologic, and surgical characteristics of the management of 15 patients (one male and 14 female) with breast desmoid tumors treated at a single institution over a 10-year period and showed that all patients underwent breast-conserving surgery with extensive resection of the tumor area and none recurred during follow-up (range 16–96 months; mean 44.86 months; median 43 months) [100]. All 15 patients had undergone surgical approaches that achieved negative margins, and none required additional surgical intervention [100]. Interestingly, these patients had comparable ages and tumor sizes to those actively monitored in the series by Duazo-Cassin [108].

Patients with progressive and/or symptomatic breast desmoid tumors should be offered surgery [2]. 

Historically, achieving tumor-free margins was considered the “Holy Grail” of any surgical approach for desmoid tumors in general and breast desmoid tumors in particular. Some studies even suggested a “safety margin” of 2–3 cm [109,110]. However, the real impact of tumor-free margins on recurrence rates is controversial, with conflicting data regarding their impact on the risk of recurrence in breast desmoid tumors and desmoid tumors in general [2,100]. The importance of negative margins in the context of breast desmoid tumors was highlighted in a study with 32 patients with surgically resected tumors, which reported a recurrence rate of 56% for margin-positive resections and 16% for margin-negative resections (although the study was underpowered to detect a statistically significant difference between both) [2,111]. Indirect evidence from other series and studies, including patients with desmoid tumors from different topographies, provided additional conflicting evidence. One series included 426 patients with extra-abdominal tumors (119 with truncal desmoid tumors), of whom 110 underwent an R0 resection and 107 underwent an R1 resection [2,111]. No significant differences were found between the R0 and R1 groups in 5-year PFS, but patients who underwent an R2 resection displayed significantly lower 2- and 10-year PFS (as low as 43% and 22%, respectively) [2,112]. Another series included 177 patients with desmoid tumors who underwent an R0 or R1 resection and showed that, although R0 was an independent predictor of PFS, patients who underwent an R1 resection still had an impressive 10-year PFS of 52% [2,113]. 

Overall, an R0 resection is recommended when technically feasible, although an R1 resection is acceptable when it allows patients to be spared significant and excessive morbidity (as may occur in the case of deeply infiltrating tumors or when resection cannot guarantee organ functionality or esthetics) [2]. 

Radiotherapy may be used as an alternative to surgery as a primary local treatment for symptomatic patients who are not ideal candidates for surgery or who have unresectable diseases [2]. 

Patients with desmoid tumors treated with radiation alone have shown interesting response rates. For example, a study of 44 patients treated with radiation showed that 91% of these patients had stable or responsive disease at 3 years and that some of these patients had durable responses to radiation beyond 3 years [2,114]. 

The use of radiation in the adjuvant setting does not appear to correlate with improved local recurrence rates after an R0 resection, but its impact on local recurrence rates after an R1 resection is less clear and appears to be antagonistic [2]. One study including 189 patients with desmoid tumors reported 10-year local recurrence rates of 27% in 78 patients with R0 resections, 54% in 40 patients with R1 resections, and 31% in 33 patients with R1 resections plus adjuvant radiotherapy [2,114]. Margin positivity was a predictive factor for local recurrence in the surgery-only group but not in the surgery plus radiotherapy group, possibly supporting the idea that radiotherapy offsets the increased risk of local recurrence in patients with an R1 resection [2,114]. On the other hand, a study of 105 patients with truncal or extremity desmoid tumors treated with surgery alone (n = 74) or surgery plus adjuvant radiotherapy (n = 31) reported no significant differences in local recurrence rates, even after controlling for margin positivity [2,115]. 

The use of adjuvant radiation appears to provide greater benefits in patients who have undergone an R1 resection, although it should be used with caution in young patients given the risk of radiation-associated sarcoma. 

As mentioned, a wide variety of systemic treatments can be used to treat unresectable breast desmoid tumors or breast desmoid tumors that recur locally despite radiotherapy and surgery [2]. In addition to TKIs and cytotoxic chemotherapy, novel agents targeting the Wnt/β-catenin and Notch pathways seem particularly promising. Typically, the use of cytotoxic chemotherapy is reserved for fast-growing symptomatic tumors, while the use of TKIs is proposed for indolent tumors, always keeping in mind the usefulness of NSAIDs in pain control [2]. 

## 10. Conclusions

Breast sarcomas, breast phyllodes tumors, and desmoid tumors are rare and potentially aggressive entities. Their prevention relies on the avoidance of known modifiable risk factors, while consistent screening strategies are advocated only for individuals with known increased hereditary risk. The genomic and mutational landscape of different sarcomas that commonly develop in the breast suggests that the mutational profile shifts as a function of previous exposure to radiation and the type and location of the sarcoma. The only significant exception to this rule seems to be the *PIK3CA* gene, whose mutations seem to be associated with sarcomas of different topographies and types, suggesting a crucial role for *PIK3CA* in different sarcomas. Breast sarcomas typically present as unilateral, painless, rapidly growing masses, occasionally with associated skin color/texture changes, and usually without nodal involvement (except for certain subtypes). A diagnosis is preferably established by breast MRI and a core needle biopsy. Staging should include a CT scan of the chest and, in addition, a CT scan of the abdomen and pelvis and a bone scan for certain subtypes (such as angiosarcoma). Breast desmoids and benign phyllodes tumors do not require additional imaging for staging. The mainstay of treatment for early-stage disease is surgical resection with negative margins (when feasible), although this is highly variable depending on the histology. The benefit and usefulness of adjuvant radiotherapy and chemotherapy are not well established and depend significantly on the histologic subtype and prior treatments. Advanced disease should be treated according to the same principles that guide the treatment of other metastatic STS, including systemic chemotherapy, radiotherapy, and pulmonary metastasectomy (for patients with isolated or limited resectable pulmonary metastases). New strategies and agents, such as targeted therapy and immunotherapy, may be used for some advanced breast sarcomas, breast phyllodes tumors, and desmoid tumors. The use of GSI in the treatment of progressive desmoid tumors is an example of an exciting new therapeutic approach that may change the treatment landscape for a specific breast tumor. Given the rarity and heterogeneity of these entities, multidisciplinarity and multi-institutional collaboration are key to designing and developing new trials that enable the generation of additional prospective data.

## Figures and Tables

**Table 1:** Summary of screening recommendations for breast neoplasms in average-risk patients using mammography. Adapted from [9,10].

	Initiation Age	Frequency
National Comprehensive Cancer Network (NCCN)	40 years old (yo)	Annual
American Cancer Society (ACS)	40–44 yo: “Qualified” 45 yo: Strong	Annual: age 40–54 yo Biennial or annual option: age > 54 yo
US Preventive Services Task Force (USPSTF)	50 yo: Grade B 40–49 yo: Grade C	Biennial
European Breast Cancer Guidelines	45–49 yo 50–69 yo 70–74 yo	Screening every 2–3 years Screening every 2 years Screening every 4 years

## Data Availability

The data can be shared up on request.
